# An Improved Map-Matching Technique Based on the Fréchet Distance Approach for Pedestrian Navigation Services

**DOI:** 10.3390/s16101768

**Published:** 2016-10-22

**Authors:** Yoonsik Bang, Jiyoung Kim, Kiyun Yu

**Affiliations:** Department of Civil and Environmental Engineering, Seoul National University, 1 Gwanak-ro, Gwanak-gu, Seoul 08826, Korea; bangys1004@snu.ac.kr (Y.B.); kiyun@snu.ac.kr (K.Y.)

**Keywords:** map-matching, Pedestrian Navigation Service (PNS), Fréchet distance, autocorrelation, Global Positioning System (GPS)

## Abstract

Wearable and smartphone technology innovations have propelled the growth of Pedestrian Navigation Services (PNS). PNS need a map-matching process to project a user’s locations onto maps. Many map-matching techniques have been developed for vehicle navigation services. These techniques are inappropriate for PNS because pedestrians move, stop, and turn in different ways compared to vehicles. In addition, the base map data for pedestrians are more complicated than for vehicles. This article proposes a new map-matching method for locating Global Positioning System (GPS) trajectories of pedestrians onto road network datasets. The theory underlying this approach is based on the Fréchet distance, one of the measures of geometric similarity between two curves. The Fréchet distance approach can provide reasonable matching results because two linear trajectories are parameterized with the time variable. Then we improved the method to be adaptive to the positional error of the GPS signal. We used an adaptation coefficient to adjust the search range for every input signal, based on the assumption of auto-correlation between consecutive GPS points. To reduce errors in matching, the reliability index was evaluated in real time for each match. To test the proposed map-matching method, we applied it to GPS trajectories of pedestrians and the road network data. We then assessed the performance by comparing the results with reference datasets. Our proposed method performed better with test data when compared to a conventional map-matching technique for vehicles.

## 1. Introduction

Navigational services have historically been used exclusively for vehicles. This situation changed in 2010, when demand for Pedestrian Navigation Services (PNS) rose due to rapid developments in smartphone and wearable technologies. Users could be informed of routes to their respective destinations and any changes in their surroundings in real time. Wayfinding and positioning benefit PNS users, but one limitation of current methods is the use of vehicle network data for these services. For the map-matching process, which is required to display GPS (Global Positioning System) points on the background road network lines, techniques designed for vehicles have been employed by most of the PNSs.

Specialized network datasets for pedestrians were recently constructed by some commercial providers. These datasets, or Pedestrian Network Data (PND), have more complex and detailed structures compared with data for vehicles. Users then can find more precise and efficient ways optimized for pedestrians. However, a problem of inaccuracy occurred when positioning or guiding users because the map-matching techniques are inherently for vehicles, and therefore are unsuitable for PND.

**Figure 1:**
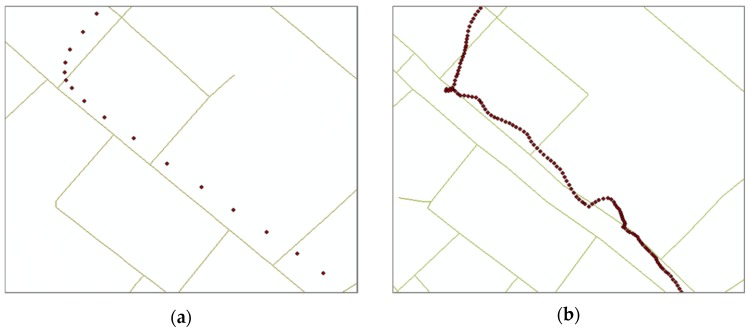
Examples of GPS signals and network datasets from (**a**) vehicle and (**b**) Pedestrian Navigation Services. Solid lines are the background network datasets, and points are the recorded GPS signals.

Moreover, pedestrians have different patterns of movements compared with vehicles. Movements of pedestrians have a much higher probability to stray from the road lines because they are much less dependent on the road lines than those of vehicles. Figure 1 shows the examples of GPS signals and network datasets. Therefore, it is difficult to predict the changes in the state of motion such as start, stop, acceleration, turn, etc. Instead, pedestrian users show relatively low velocity so that we do not need to compensate for the delay that occurred in processing time of matching. Table 1 shows the differences between navigation services for a vehicle and a pedestrian.

As shown in Table 1, we would face different kinds of challenges at a spatial scale of PNS. Because the complexity of road network data or movements of users becomes larger compared with the positioning accuracy, the distance between a user’s location and the road segment may exceed the separation between neighboring roads. GPS condition is also worse because of disturbance by buildings in urban environments [1]. Even if we derive precise locations with the help of various positioning techniques such as GPS, Wi-Fi, and Dead Reckoning (DR), there still remains the problem of inconsistency between the PND road segments and the actual location of the user.

Our objective was to match the location signals with the PND road lines in real time, rather than to identify their exact positions. Thus, we proposed a new map-matching technique that can be applied to PNS, considering the features of pedestrian movement and PND. We assumed our system utilizes the location signals obtained from the GPS module. The rest of this paper is set out as follows. Section 2 summarizes related works about map-matching and positioning techniques. Section 3 describes the theoretical basis of the Fréchet distance approach for matching linear spatial datasets. Section 4 presents a modified method for real-time map-matching that is appropriate for the PNS. Section 5 shows the experimental results and assessment through comparison with the conventional approach. Section 6 gives our conclusions.

## 2. Literature Review

### 2.1. Map-Matching Techniques

Many reports in the literature detail map-matching methods for vehicle navigation systems. These studies flourished with the rapid development of the Intelligent Transportation System (ITS) and telematics technologies in the 2000s. Most of these methods estimated the precise location of a vehicle using a GPS signal and auxiliary techniques such as Dead Reckoning (DR). The Kalman Filter method has generally been used and improved by the extensions and modifications.

Bernstein and Kornhauser [2] introduced various algorithms for map-matching of personal navigation devices. This study investigated the theoretical basis for research about map-matching techniques of car navigation system. Quddus et al. [3] applied the Extended Kalman Filter to the GPS signal for map-matching of the vehicle navigation systems. Velaga et al. [4] presented a topological map-matching method based on weights from the attributes of road network data. They estimated the position by DR in addition to the GPS signal. These map-matching methods focused on the limitation of GPS signals falling behind rapidly-moving vehicles. Various techniques were presented to compensate for the delay in processing GPS signals, e.g., estimating the future location signals using filters for combination with the DR.

By contrast, the major PNS limitation is that the positional error of GPS exceeds the location range estimated by the velocity of a moving pedestrian. For example, if a user moves 1 m every second and the positional error is 5 m, the direction of the trajectory becomes uncertain over a short distance. This problem is originated from (1) frequent changes of direction, (2) close distance from buildings or trees which could disturb the GPS signals, and (3) the high complexity of the PND.

### 2.2. Map-Matching Techniques for the PNS

A map-matching technique for PNS is needed to focus on correcting current location with a positional error larger than the actual movement of a user, whereas the vehicle navigations focus on estimating a future location. In addition, a technical improvement is also needed for more complicated and detailed road networks, as the PND is being constructed in urban areas.

Many studies have been conducted on positioning techniques for pedestrian users. Some researchers used filters to improve performances of map-matching. Martin et al. [5] tested various filters generally used for map-matching problems of GPS trajectories. Jirawimut et al. [6] combined GPS and DR with corrected parameters to improve the accuracy of positioning. Other researchers utilized additional sensors for more precise positioning. Sabatini [7] provided a DR-based method for PNS using auxiliary measurements such as inertial and magnetic sensors. Ren and Karimi [8] identified types of walking using inertial and magnetic sensors as well as GPS signals. The authors split the trajectories based on different walking types and matched each segment to the PND. Indoors, a Wi-Fi signal becomes useful for positioning. Wilk and Karciarz [9] matched the indoor tracking data using Wi-Fi and DR with the PND.

Recently, 3D-model-based positioning methods for urban area are being studied by many researchers. Kumar and Petovello [10] used a 3D city model and Betaille et al. [11] used 3D urban digital map data to model how the signal is affected by buildings and improve the accuracy of the GNSS positioning method. Hsu et al. [12] tested the 3D-model-based positioning method by applying location signals obtained from many kinds of Global Navigation Satellite System (GNSS) sensors. Groves et al. [13] presented current challenges in 3D map-matching.

Although there are plenty of techniques for matching the trajectory of pedestrian to network data, most of these methods focus on matching a complete trajectory or a part of the path. Since these techniques are not real-time methods, they are not appropriate to be applied to the Location Based Services (LBS). Other researchers studied real-time positioning techniques for pedestrians [6,7]. However, no consideration was given in those studies to pedestrian network data that are characterized by detailed features of roads.

### 2.3. Objectives of the Research

In this paper, we propose a new map-matching technique suitable for PNS. We hypothesized that this method would provide (1) real-time matching and relocation of an input GPS signal and (2) higher accuracy of map-matching for more detailed road networks compared with the positional error of the GPS signals.

To achieve this goal, we adopted the concept of the Fréchet distance, one of the measures of geometric similarity between two linear features. The Fréchet distance is appropriate for the trajectories of moving objects because it regards a linear feature as a location function of the time variable. We reviewed the underlying theory of a matching method based on the Fréchet distance. Then we made some improvements to propose a new map-matching method between the GPS signals of pedestrian and the PND.

## 3. Theoretical Bases of Line Matching using the Fréchet Distance Approach

### 3.1. Fréchet Distance Concepts

The Fréchet distance was originally proposed by Fréchet [14] as a measure of distance between two curves. It was used to identify the geometrical similarity between linear features in the field of computational geometry and many other fields. The Fréchet distance between two curves is defined as below.

**Definition**: *Let* $f:\left[a,a\prime \right]\to {\mathbb{R}}^{2}$ *and* $g:\left[b,b\prime \right]\to {\mathbb{R}}^{2}$ *be curves. Then* $\delta_{F}\left(f,g\right)$ *denotes their Fréchet distance, defined as*:

(1) $$\delta_{F}∶=\underset{\begin{array}{c} \alpha \left[0,1\right]\to \left[a,a^{\prime }\right]\\ b\left[0,1\right]\to \left[b,b^{\prime }\right] \end{array}}{\inf}\underset{t\in \left[0,1\right]}{\max}\|f\left(\alpha \left(t\right)\right)-g\left(\beta \left(t\right)\right)\|,$$

*where, ‖.‖ denotes the* $L_{2}$ *norm, and* α *and* β *range over continuous and increasing functions with* α(0)=a*,* α(1)=a′*,* β(0)=b*, and* β(1)=b′ *only.*

A popular illustration of the Fréchet metric shows a man walking his dog. He is walking on one curve and the dog is walking on the other curve. Both subjects are allowed to control their speed but are not allowed to go backwards. The Fréchet distance between the curves is the minimal length necessary for a leash [15].

**Figure 2:**
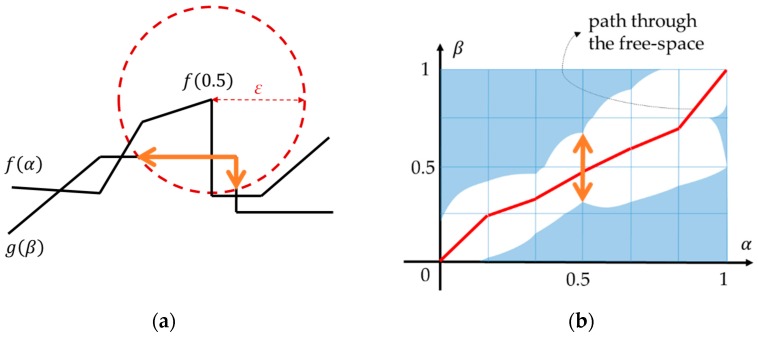
An example of (**a**) two curves f and g and (**b**) their free-space diagram for distance threshold ε. Two curves were parameterized with α and β. The thick arrowed lines are the part of g within distance ε from f(0.5). There exists a path, depicted as a thick solid line, through the free space in the diagram [15].

The free-space diagram, a graphical technique for computing the Fréchet distance, was proposed by Alt and Godau [15]. They parameterized two curves f and g with time variables α(t) and β(t), respectively. Then the free space between two curves for a given distance threshold ε is defined as a two-dimensional region in the parameter (α, β) space that consists of all point pairs on the two curves at a distance of not more than ε. Figure 2 gives an example of two curves and their free-space diagram. The white area in the diagram refers to the free space, which is defined as in Equation (2) [15].

(2) $$D_{\varepsilon }\left(f,g\right)∶=\left\{\left(\alpha ,\beta \right)\in {\left[0,1\right]}^{2}|d\left(f\left(\alpha \right),g\left(\beta \right)\right)\leq \varepsilon \right\}$$

Here, $d\left(P_{1},P_{2}\right)$ means the Euclidean distance between two points $P_{1}$ and $P_{2}$.

The Fréchet distance F(f,g) is at most ε if and only if the free space $D_{\varepsilon }\left(f,g\right)$ contains a path from the lower left corner to the upper right corner, which is monotone both in the horizontal and in the vertical direction.

### 3.2. Fréchet Distance for Curve-Matching Problem

The Fréchet distance can be applied to curve-matching problems because the method provides geometrical similarity between two linear features. In Figure 2b, any pair (α, β) on the shortest path from the lower left corner to the upper right corner may be a matching pair of two curves. Some work has been done to apply and develop the approach.

Alt et al. [16] proposed a matching algorithm using the Fréchet distance between two curves and provided a mathematical proof. Based on the proof, Efrat et al. [17] proposed the polygon sweeping method, and Devogele [18] merged two polylines for vector data integration. These works assumed the cases of matching two single curves. However, the road network dataset consists of multiple line objects connected each other. To extend the Fréchet distance approach to the one-to-many matching, some works have been done to modify or improve the approach.

**Figure 3:**
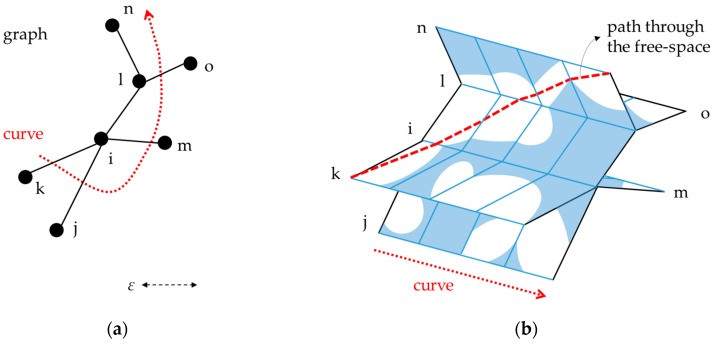
An example of (**a**) a trajectory curve and a graph and (**b**) an extended free-space diagram between them. The shortest path (a thick dashed line in the diagram) becomes the matching result (from [19,20]).

Alt et al. [19] extended the free-space diagram to match a curve to the “graph” consists of edges and nodes. The free-space diagram is drawn as Figure 3 below, a set of multiple diagrams orthogonal to the plane of the graph. The shortest path from the start to the end becomes the matching result of the curve and the graph.

Brakatsoulas et al. [20] applied this approach to the matching of a vehicle GPS trajectory with the road network. Its performance was assessed and compared with the incremental map-matching method. They showed that the Fréchet-distance-based method produced a better matching result. The Fréchet-distance-based method is appropriate for matching a trajectory with road network data because it considers the sequence and continuity of a curve when evaluating the matching process.

### 3.3. Limitations of the Fréchet-Distance-Based Approach

The approaches described earlier assumed that a complete trajectory was recorded before the matching process. However, map-matching should be delivered in real time to a user at the current location. The algorithm of the Fréchet-distance-based method should be modified to process the GPS signal point-by-point in order to apply the procedure to map-matching.

Moreover, the Fréchet-distance-based method uses a single value of the distance threshold ε for a curve. However, GPS signals are accompanied with various types of positional error. Especially in case of pedestrians the error seems to be more irregular and unpredictable than vehicles. If there are some parts where the error inflates instantaneously, the threshold ε becomes large for the trajectory due to the part with the maximum positional error. This results in an uncertainty of matching, and consequentially a low performance.

**Figure 4:**
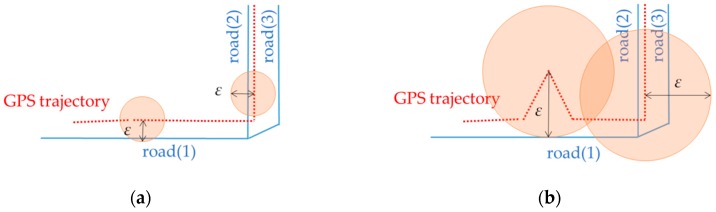
The Fréchet distance approach may be affected by the positional error: (**a**) An ideal case: We can identify the set of matched roads with a small value of threshold ε; (**b**) a realistic case: The positional error inflates at some parts and ε becomes larger due to errors.

In Figure 4, for example, if the positional error remains steady (Figure 4a), a small value of the threshold is enough to match the whole trajectory. If the positional error inflates instantaneously in some parts of the trajectory (Figure 4b), the threshold value would become larger to continue matching the error parts. This leads to uncertainty in the matching results because there are multiple solutions in other parts of the trajectory.

## 4. A Real-Time and Adaptive Map-Matching Method

In this paper, we made modifications with the Fréchet-distance-based approach to overcome the limitations. Our new method could deliver map-matching results in real time. The method also reduced matching uncertainty by allowing flexible variations in the threshold in response to the positional error of GPS signals.

### 4.1. Modification for Real-Time Map-Matching

The Fréchet-distance-based matching method is basically applied to a complete trajectory. We modified the algorithm into a point-by-point process by drawing free-space diagrams at every single point of the input GPS signal. Each point is then map-matched on the road network immediately.

**Figure 5:**
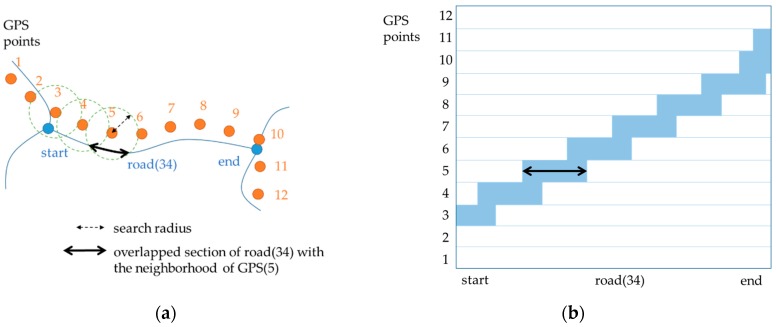
An example of (**a**) GPS signal and a road segment and (**b**) a free-space diagram from the example. For example, an element of the free space at the 5th GPS point is depicted as a bar corresponding to an overlapped section of the road segment and a search range of the point.

A free-space diagram drawn by the existing Fréchet distance method (Figure 2) can be transformed into the diagram in Figure 5 by replacing f by the road segments and g by the discrete GPS signal. The free space is then composed of the overlapped sections between road segments and search ranges of GPS points. The Fréchet distance is the minimum value of the search radius to make the free space contain a path from the lower left corner to the upper right corner.

Because we cannot estimate the Fréchet distance without any information about future locations, we first determined the initial value of the search radius and increased gradually until we reached the maximum positional error. The initial value was set empirically to be a larger value than the shortest distance to the nearest road segment. For the first point, we clipped out the overlapped section from the road segment and drew a bar-shaped entity at the corresponding location in the diagram space.

This procedure continued until the next GPS point. For each point, we check whether its overlapped section is connected with the previous one. If not, the search radius is then increased to make the corresponding free space connected. Let $D_{min}\left(i\right)$ be the shortest distance between the i-th GPS point and the previous overlapped section; the search radius R(i) is determined as a larger one between R(i−1) and $D_{min}\left(i\right)$ (Equation (3)). This is because $D_{min}\left(i\right)$ is the least value for the search radius needed to maintain the connectivity of the free space.

(3) $$R\left(i\right)=\max\left\{R\left(i-1\right),\;D_{min}\left(i\right)\right\}$$

**Figure 6:**
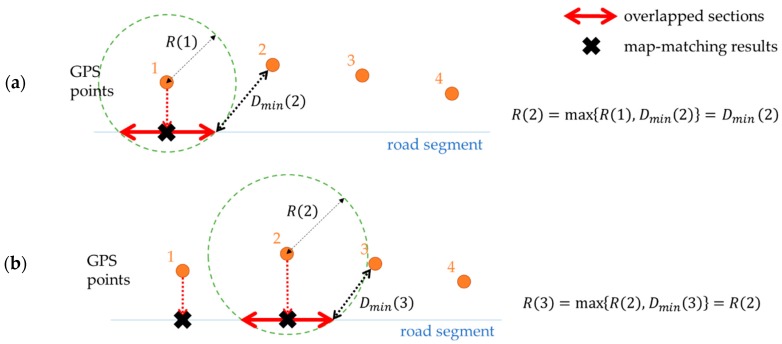
Determining search radii and relocating GPS points: (**a**) Search radius for the 2nd point is $D_{min}\left(2\right)$ because $R\left(1\right)<D_{min}\left(2\right)$; (**b**) search radius for the 3rd point is R(2) because $R\left(2\right)>D_{min}\left(3\right)$. Map-matching results are identified to the geometric centers of the overlapped sections.

The GPS point location is then moved to the corresponding overlapped section of the road segment. The geometric center of the overlapped section becomes the location for the next move. In Figure 6, x-shaped points on the road segments are the results of map-matching of individual GPS points.

### 4.2. Modification for Adaptive Map-Matching

As we mentioned above, the map-matching results may be globally affected by the maximum positional error. To overcome this limitation, we made the search range “adaptive” to changes in the positional error of the GPS signal. Adaptation can be made for two aspects of the search range—the search center and the search radius. First, a center of a search range can be adapted according to the direction of the positional error. Second, a radius of a search range can also be adapted according to the size of the positional error. With the help of such adaptations, the matching result can become more precise as the search range is narrowed down.

The amount of an adaptation can be determined by the auto-correlation between positional errors of two consecutive GPS points. This is because a large correlation creates a high similarity between map-matching results. Location information recorded by GPS contains the positive correlations among simultaneous observations [21]. Major error sources are satellite orbit, troposphere, multipath, antenna phase, and centering for the correlation in a short time range of the signal.

**Figure 7:**
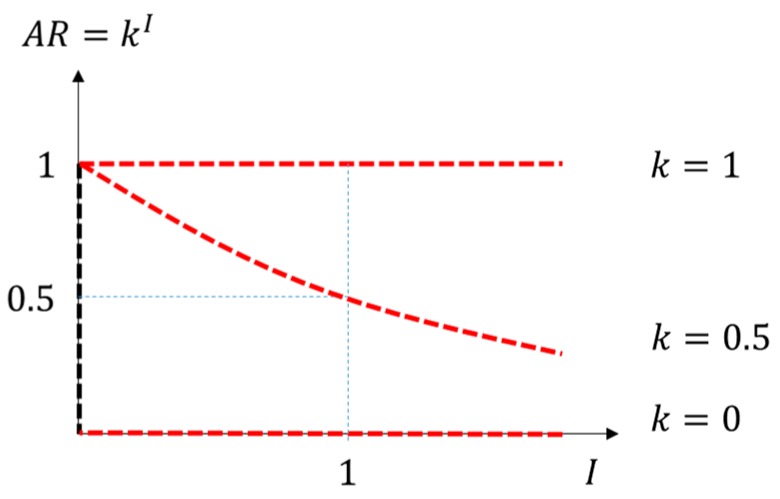
Relation between the Adaptation Ratio and the GPS point gap. If the Adaptation Coefficient k equals 1, AR remains 1 regardless of the point gap. If k equals 0, AR becomes 0 for all nonzero values of the point gap.

However, it is impossible to model every environmental factor of error sources used to estimate the auto-correlation coefficient. Instead, we assumed the Adaptation Ratio (AR), representing an amount of correlation varying with the distance between points. The AR decays exponentially according to the distance between two consecutive points (Equation (4)):

(4) $$AR\left(i\right)=k^{I\left(i,i-1\right)},$$

where k denotes the Adaptation Coefficient, representing an amount of correlation between two GPS points, and I(i,i−1) denotes the distance between the i-th and (i−1)-th point of a GPS trajectory, which was standardized by dividing by the average point gap. The AR function in relation to I is depicted in Figure 7. AR is close to 1 when the point gap is close to 0, and converges towards 0 as the point gap gets larger. As we assumed the AR to decay exponentially, k should be within the range of 0≤k≤1. The smaller k value we set, the faster the AR decreases.

The Adaptation Coefficient k is determined to a value between 0 and 1, representing the homogeneity of positional error. If k is close to 1 the positional errors of points are relatively homogeneous. Then the search range is adapted to be similar to that of the previous point. On the other hand, if k is close to 0 the positional errors are independent. Then the search range is irrelevant to the previous point. Center of the search range of the i-th point, or $\vec{C\left(i\right)}$ is determined by Equation (5) below:

(5) $$\vec{C\left(i\right)}=\vec{P\left(i\right)}+\vec{disp\left(i-1\right)}\cdot k^{I\left(i,i-1\right)},$$

where $\vec{P\left(i\right)}$ denotes the location of the i-th GPS point and $\vec{disp\left(i\right)}=\vec{M\left(i\right)}-\vec{P\left(i\right)}$ is a vector consisting of the distance and direction of the map-matching result of the i-th point.

The radius of the search range of the i-th point is determined via Equation (6) below:

(6) $$R\left(i\right)=\max\left\{R\left(i-1\right)\cdot k^{I\left(i,i-1\right)},\;D_{min}\left(i\right)\right\}.$$

Assuming that the positional error is independent (k=0), the search radius at a point equals $D_{min}\left(i\right)$, the least value for the search radius to maintain continuity of map-matching. In this case, the uncertainty can be reduced considerably because the search radius is minimized at each point of the trajectory. Instead, it may raise the possibility of no correct match within a range of search radii. On the other hand, if k=1, the positional error is assumed to be homogeneous through the trajectory. Therefore, a small part of the maximum positional error can make the search radius increase too much for the rest of the trajectory. This may cause uncertainty in map-matching.

**Figure 8:**
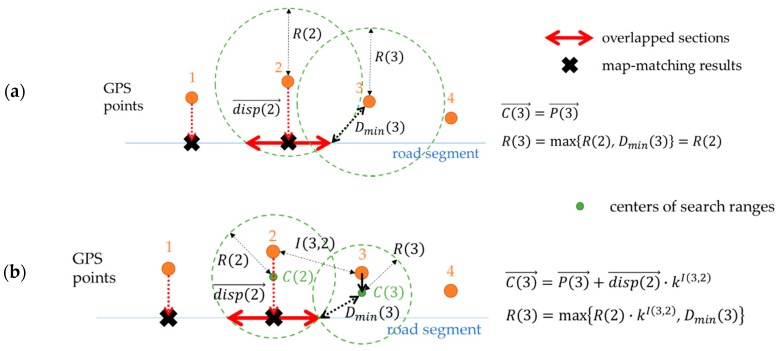
Determining search radius with adaptation when the positional error decreases $\left(D_{min}\left(i\right)<R\left(i-1\right)\right)$: (**a**) Assuming no adaptation (the basic method in Section 4.1.), R(i)=R(i−1) and $\vec{C\left(i\right)}=\vec{P\left(i\right)}$, same as the Figure 6; (**b**) Assuming adaptation with a certain coefficient (0≤k≤1), $D_{min}\left(i\right)\leq R\left(i\right)<R\left(i-1\right)$, which means that search radius shrinks and the search center is displaced by $\vec{disp\left(i-1\right)}\cdot k^{I\left(i,i-1\right)}$.

For an example of k=0.5, as shown in Figure 8, the search range of the i-th GPS point is translated in the direction of the map-match of the previous point. Its radius also decreases in proportion to $k^{I\left(i,i-1\right)}$ by Equation (6).

Table 2 summarizes how to determine the search radius and center for a GPS point, depending on the characteristic of positional error. We can adjust the k value to determine the proper ratio between the two sides. The characteristic of the positional error is difficult to estimate as the GPS signal is affected by the user’s environment and movements. Therefore, we determined a proper k value in an empirical way.

### 4.3. Detecting and Correcting Errors Using a Reliability Index

In a map-matching problem, positional or topological errors of the road network dataset also exist, as well as errors in GPS signals. Such inherent errors cause some wrong matches even though the aforementioned map-matching process works well. These wrong matches lead to the map-matching onto a wrong road segment or moving in the wrong direction.

To solve this problem, we added a process of evaluating a geometric reliability at each matching result in the trajectory. The Reliability Index (RI) is defined by Equation (7), an angle difference between directions of the GPS trajectory and its map-matched result. RI is calculated as a cosine of the angle difference so that the value is located between −1 and 1. A larger value of RI means a lower possibility that the corresponding GPS point was matched to the wrong position.

(7) $$RI\left(i\right)=\cos\theta \left(i\right)=\frac{\vec{P\left(i-1\right)P\left(i\right)}\cdot \vec{M\left(i-1\right)M\left(i\right)}}{\left|\vec{P\left(i-1\right)P\left(i\right)}\right|\left|\vec{M\left(i-1\right)M\left(i\right)}\right|},$$

where, θ(i) denotes the angle between the trajectory and the direction of map-matching result at the i-th point of the GPS signal, $\vec{P\left(i\right)}$ denotes the i-th point, and $\vec{M\left(i\right)}$ denotes the map-matched position corresponding to the i-th point.

**Figure 9:**
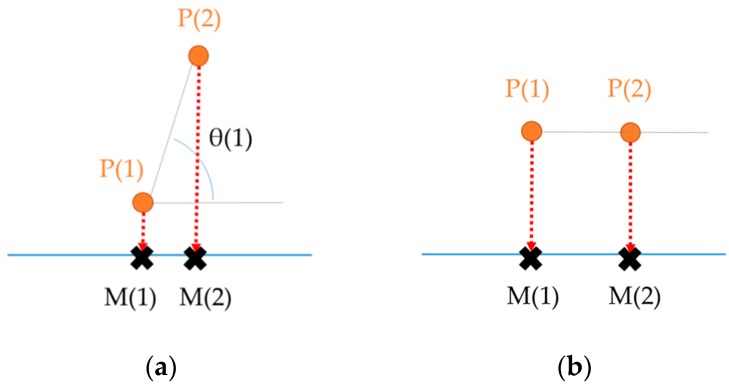
Visual explanations of reliability of a map-matching result: (**a**) unreliable match (cosθ is close to or less than 0); (**b**) reliable match (cosθ is close to 1) for the 2nd point compared with the matching result of the previous point.

A visual explanation of the reliability of local map-match results is given in Figure 9. If the angle between the trajectory and the map-matched result is large, the result seems to be unreliable. An angle larger than 90° also implies an unreliable match because it means that the point was matched in the wrong direction. On the other hand, the closer the angle is to 0, the more reliable the match result would be.

A map-matching result with an RI lower than the certain threshold value should be regarded as an unreliable match and excluded from the result data. The larger threshold leads to the higher precision but the lower recall for map-matching, and vice versa. The proper value of the threshold was determined by statistical analysis using some training data.

## 5. Results Using Test Data

### 5.1. Data Used

The adaptive Fréchet-distance-based approach we proposed in Section 4 was tested. We determined a test site in Seoul and obtained some GPS trajectories of pedestrians. A set of PND (Pedestrian Network Data) that consist of links (road segments) and nodes (endpoints of links) was used as the background road network. This dataset is describing various kinds of paths for pedestrians including sidewalks, crosswalks, and pathways. It was constructed based on the 1:1000-scaled national digital map, which has a maximum horizontal error of 0.7 m according to the accuracy standards of South Korea [22].

**Figure 10:**
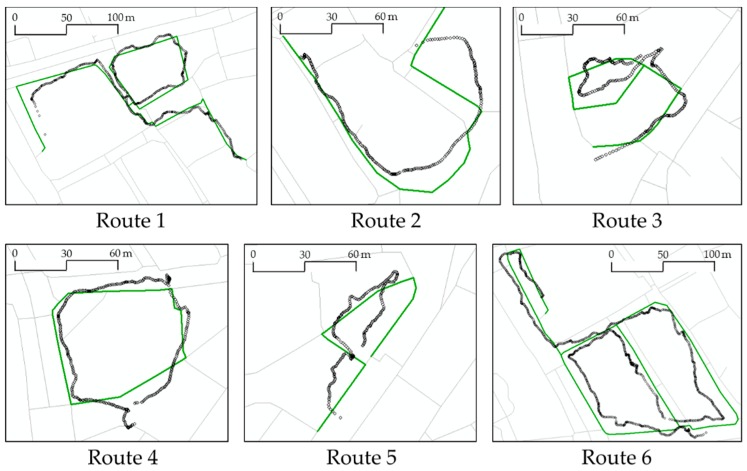
Six planned routes (thick parts from the background PND) and collected GPS trajectories along the routes (small circles).

The test site covers an area of approximately 0.14 km^2^ and is located in Jung-gu, Seoul Metropolitan City. We planned six routes arbitrarily along the roads in the area. Then we walked along those routes to collect the GPS signals of the trajectories. The lengths of the routes were determined by considering the walking distance of the “short-range” pedestrians. We used a GPS sensor embedded in a smartphone with an Android OS. The time interval of the recording was set to 1 s and the minimum displacement between points was not limited. The GPS trajectories and their planned routes (ground truth) are depicted in Figure 10. Specifications of the obtained datasets are summarized in Table 3 below.

### 5.2. Pre-Processing for Application of the Methodology

**Figure 11:**
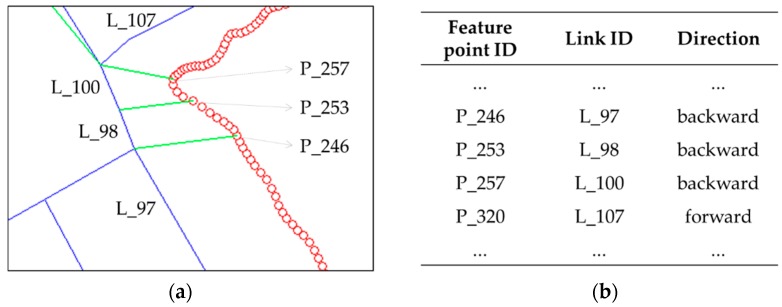
A part of the ground truth dataset (**a**) and its matching table (**b**), identified manually for selected feature points from Route 6. Thick lines are the PND links and circles are the GPS-recorded points. Lines connecting points and PND are the reference matches of the ground truth dataset. In this example, points between P_246 and P_253 should be matched to PND link L_98 and the direction of the link is opposite to that of the GPS signal.

For each GPS trajectory, the ground truth dataset was constructed based on the actual positions where the signals were recorded. Because we cannot identify the actual positions for every points of the trajectory, we selected some feature points—mostly intersections—that are easy to identify. Using the time log of those feature points, we can identify the actual positions on PND where the points should be matched. Then we can construct information about the matching pairs of all GPS points and their corresponding road segments. Such ground truth data are enough to assess the match results because the intersections are more important than the other parts for map-matching. Figure 11 depicts a part of the ground truth dataset and its matching table, constructed manually for Route 6.

**Figure 12:**
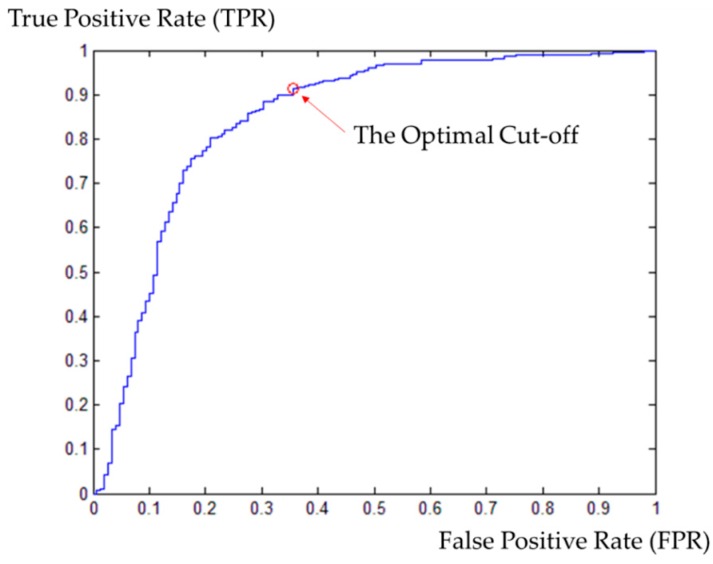
The ROC curve from the training dataset for the Fréchet-distance-based method. The relationship between TPR and FPR is depicted and the optimal cutoff on the curve is determined to be the point (0.3557, 0.9140).

Then, we employed the GPS trajectory of Route 1 as a training dataset to determine the threshold of RI described in Section 4.3. The training dataset was map-matched with the PND using the Fréchet-distance-based method. The matching result of each point can be identified as correct or wrong by comparing it with the ground truth dataset. Then we plotted a Receiver Operating Characteristics (ROC) curve using the RI values of the matching results. A ROC curve is a technique for selecting a proper criterion for a classification problem, based on its performance [23]. It depicts the relative tradeoffs between benefits (true positive rate, TPR) and costs (false positive rate, FPR). Figure 12 shows a ROC curve derived by map-matching for the training dataset.

As shown in Figure 12, the point at (0.3557, 0.9140) on the ROC curve is the closest weighted point to (0,1), or the ideal classifier. Then the classifier’s best accuracy occurs at a threshold of 0.7301. Therefore, we classified a matching result with an RI lower than 0.7301 as an unreliable match and excluded it from the result data. The Area under the Curve (AUC) for the ROC curve is 0.8452. This value indicates that our RI is a good measure for identifying wrong matches.

### 5.3. Performance Indices for Map-Matching Methods

The performance of the method was evaluated by two indices: (1) Ratio of Correct Match (RCM) and (2) Average Positional Error (APE). RCM is computed by dividing the number of points matched onto the correct links by the total number of matched points (Equation (8)). A correct link for each point can be identified because the ground truth dataset was constructed at feature points including intersections. RCM is then evaluated by comparing the matching with the ground truth dataset.

(8) $$RCM=\frac{n_{CM}}{n_{M}},$$

where $n_{CM}$ denotes the number of points matched onto the correct links and $n_{M}$ denotes the total number of matched points.

**Figure 13:**
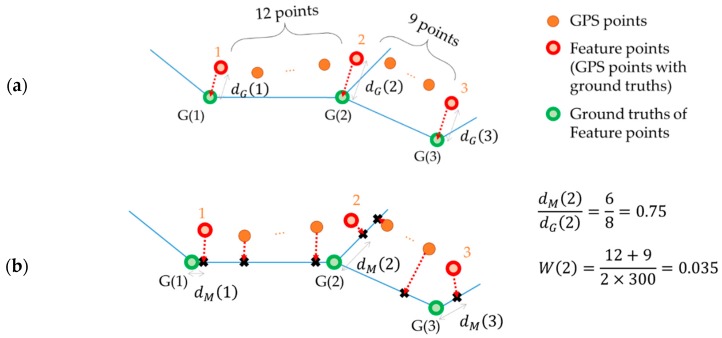
A visual explanation for calculating APE: (**a**) The ground truth tells us that FP(2) should be map-matched to G(2) and there are 12 points before and nine points after the FP(2). Then W(2) is calculated as $\frac{12+9}{2\times 300}=0.035$. (**b**) The map-matching result of FP(2) derived by the map-matching method is different from G(2). A positional error of FP(2), or $d_{M}\left(2\right)$ is then standardized by dividing it by $d_{G}\left(2\right)$, that is, $\frac{d_{M}\left(2\right)}{d_{G}\left(2\right)}=\frac{6}{8}=0.75$.

APE is an index for measuring the difference between the map-matched position and the ground truth dataset. The differences can be evaluated only at the feature points because the ground truth data were constructed only for the feature points. For each feature point, therefore, we calculate a weighting based on its ratio to the whole trajectory. We then computed an average of differences at every feature points using their weightings. As an example, in Figure 13, among the 300 recorded GPS points in the trajectory, there are 12 points before and nine points after Feature Point 2 (FP(2)). Weighting for FP(2) is then calculated as $\frac{12+9}{2\times 300}=0.035$. Furthermore, the differences at feature points were standardized by dividing them by the match distances derived from the ground truth data. In the example in Figure 13, the difference evaluated at the feature FP(2) is 6 m and its corresponding match distance derived from the ground truth data is 8 m. Then the difference value is standardized as $\frac{6}{8}=0.75$. The APE of the whole trajectory is evaluated by Equations (9) and (10) below:

(9) $$APE=\sum_{j=1}^{n_{FP}}W\left(j\right)\cdot \frac{d_{M}\left(j\right)}{d_{G}\left(j\right)},$$

where $n_{FP}$ denotes the total number of feature points in the ground truth dataset for a trajectory, W(j) denotes the weighting for the j-th feature point, $d_{M}\left(j\right)$ denotes the difference between the map-matched position and the ground truth for the j-th feature point, and $d_{G}\left(j\right)$ denotes the match distance derived from the ground truth data for the j-th feature point. W(j) is calculated by Equation (10) below:

(10) $$W\left(j\right)=\frac{n_{j-1,j}+n_{j,j+1}}{2n_{P}},$$

where $n_{j-1,j}$ denotes the number of GPS points between the (j−1)-th and j-th feature point and $n_{P}$ denotes the total number of GPS points in a trajectory.

### 5.4. Application and Assessment of Proposed Method

For each GPS trajectory, the proposed method was applied to solve the map-matching problem with the background PND. The Fréchet-distance-based map-matching algorithm was implemented in Matlab 8.3 (MathWorks Inc., Natick, MA, USA) and tested using a PC with i7 processor (Intel, Santa Clara, CA, USA) 2.7GHz.

For Trajectory 1, we first applied the adaptive Fréchet-distance-based method for map-matching with the PND. By increasing the adaptation coefficient k from 0 to 1 by 0.1, we evaluated RCM and APE and observed how they change as k changes.

**Figure 14:**
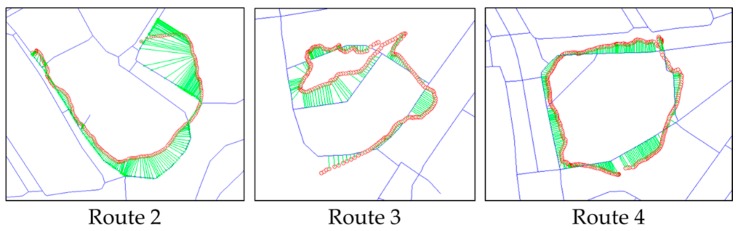
Some parts of the map-matching results obtained by the adaptive Fréchet-distance-based method. Thick lines are PND segments and circles are GPS-recorded points. Lines connecting points and PND segments are the matching relations from the results.

As shown in Table 4, RCM and APE both showed the best performance with a k value of about 0.2. Therefore, we set k=0.2 in map-matching experiments for the rest of the collected GPS trajectories. For the rest of the five trajectories, we applied the adaptive method with the parameters determined above. Then we assessed their results using RCM and APE. The performance indices derived from the results are shown in Table 5. Average Fréchet distance in the Table 5 means an average of distances between GPS points and their map-matched positions. Figure 14 shows some parts of the map-matching results by the adaptive method.

**Figure 15:**
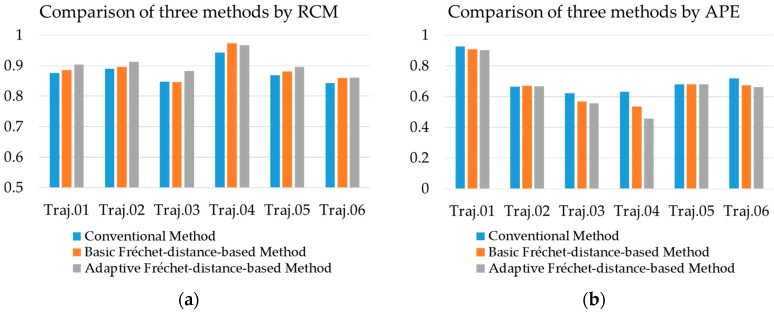
Comparison of performances of three map-matching methods–Conventional, Basic, and Adaptive Fréchet-distance-based method: (**a**) Comparison by RCM; (**b**) comparison by APE.

To check the feasibility of our methodology, we also tested a conventional map-matching method used by vehicle navigation services and compared its results with those of our methodology. Here, a map-matching method from Yang et al. [24] was applied to the same datasets and assessed by the same performance indices. This method is suitable for the map-matching of rapidly moving vehicles because it was modified for GPS signals with long time intervals. In addition, the basic (“non-adaptive”) Fréchet-distance-based method was also tested and compared with the adaptive method we proposed. Table 6 shows the performance indices resulted from two existing map-matching methods. We compared these results with those from the adaptive method in Figure 15.

**Figure 16:**
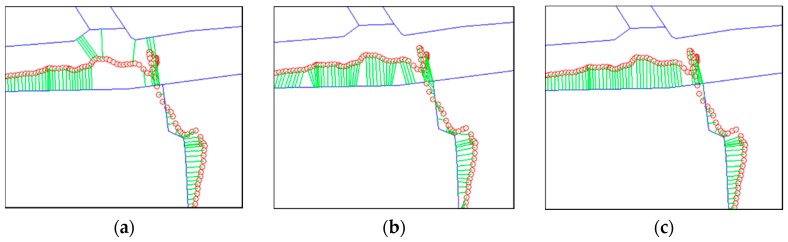
Comparison of results from (**a**) conventional method, (**b**) basic Fréchet-distance-based method, and (**c**) adaptive Fréchet-distance-based method applied to Trajectory 4. Thick lines are PND segments and circles are GPS-recorded points. Lines connecting points and PND segments are the reference matches of the ground truth dataset. Applying the Fréchet-distance-based method reduced the error and increased the number of correct matches.

Considering the RCM, the adaptive method provided 2.625% higher value on average than the conventional method. This result implies that the effect of the Fréchet distance approach exists. Moreover, it was 0.866% higher than the basic Fréchet-distance-based method. This is because the adaptive method may reduce the uncertainty of map-matching and lead to a decrease in incorrect matches. For example, for Trajectory 4, the number of incorrect matches decreased, as we see in Figure 16. Considering the APE, the adaptive method provided 9.604% lower value on average than the conventional method, and 3.572% lower than the basic Fréchet-distance-based method. Results in APE also showed that the proposed method provided better performance.

In Trajectories 2 and 3, because the positional error inflates in some parts, the basic Fréchet-distance-based method did not provide higher performance than the conventional method. Otherwise, the adaptive Fréchet-distance-based method provided higher performance than other methods. These cases showed that the adaptive method is less affected by the positional error.

In the case of the Trajectory 4 (Figure 16), RCM was higher than other trajectories because its GPS signal had a smaller positional error. Two Fréchet-distance-based methods provided better performance than the conventional method. However, there was no notable effect on RCM of improvement via the “adaptive” method because the positional error did not fluctuate irregularly. Instead, there is a significant improvement in APE due to the adaptive search radius.

Trajectories 5 and 6 have relatively irregular positional error. Considering the RCM, both of the Fréchet-distance-based methods provided relatively good results. We can see that the Fréchet-distance-based approach considers continuity in the map-matching process. Considering the APE, although the results were better than the conventional method, the differences were not so significant. This is because calculating APE does not reflect the continuity of individual points in a trajectory.

Considering the characteristics of PNS–the more complex and detailed network data and the more irregular movement of pedestrians—this result tells us that the Fréchet-distance-based approach is more suitable for PNS.

## 6. Conclusions

Pedestrian Navigation Services (PNS) may be used for various patterns of movements made by pedestrian users. Moreover, the Pedestrian Network Data (PND) have more complex and detailed structures compared with similar datasets for vehicles. Therefore, a specialized technique for map-matching is required that considers the characteristics of pedestrians and the PND.

In this paper, we proposed a new map-matching methodology that is based on the Fréchet distance, the measure of the similarity between two linear features. We first reviewed the theoretical basis of the Fréchet distance approach for matching linear spatial datasets. In addition, we applied the approach to the map-matching problem between GPS trajectory and PND. Although the approach can provide the matching results, reflecting the connectivity of the network data, it is affected considerably when the positional error of the GPS signals fluctuates irregularly.

To overcome this, we modified the method to be adaptive to the positional error of GPS signals. The adaptation was made for the search center and the search radius at each GPS point. The Adaptation Ratio (AR) was determined by an “Adaptation Coefficient,” which was based on the auto-correlation of the consecutive GPS points. At each point of map-matching, we computed the Reliability Index (RI) to filter out the wrong matches.

Then we applied our adaptive Fréchet-distance-based method to some sample datasets and assessed its performance by two indices: Ratio of Correct Matches (RCM) and Average Positional Error (APE).

Results from the adaptive method showed performances of 0.8910 in RCM and 0.6198 in APE. To check the feasibility of the proposed method, we tested the conventional map-matching method for vehicles and the “basic” (non-adaptive) Fréchet-distance-based method. As a result, the adaptive method showed better performances than the conventional method (0.8682 in RCM and 0.6838 in APE) and the basic method (0.8836 in RCM and 0.6403 in APE). We also noticed that our adaptive Fréchet-distance-based method is more suitable for PNS because it provided higher performance for the sample GPS datasets with larger positional errors.

Our proposed map-matching technique for tracking PNS users may improve the accuracy of future studies focusing on a determination of the adaptation coefficient or evaluating bigger datasets.

## Figures and Tables

**Table 1:** Differences between navigation services for vehicles and pedestrians.

Feature	Vehicle Navigation Services	Pedestrian Navigation Services
Movement	fast, predictable	slow, hard to predict
Road network	simple, generalized	complex, detailed
GPS conditions	relatively far from buildings	easily disturbed by buildings
Modifiability	hard to get back to the planned path	easy to reroute when lost
Operation	hard to operate while driving	easy to operate while walking

**Table 2:** Calculating the search radius and center for a GPS point according to the characteristic of positional error.

Positional Error	Independent	...	Homogeneous
Adaptation Coefficient (k)	0	...	1
Search radius	$R\left(i\right)=D_{min}\left(i\right)$	...	$R\left(i\right)=\max\left\{R\left(i-1\right),D_{min}\left(i\right)\right\}$
Search center	$\vec{C\left(i\right)}=\vec{P\left(i\right)}$	...	$\vec{C\left(i\right)}=\vec{P\left(i\right)}+\vec{disp\left(i-1\right)}$

**Table 3:** Planned routes and collected GPS trajectories. Average errors were calculated from the recorded accuracy values of GPS points.

Planned Routes	Collected GPS Trajectories			Average Error (m)
	No. of Points	No. of Feature Points	Total Length (m)	
1	579	23	807.6	18.30
2	254	15	370.6	22.25
3	246	13	359.4	11.65
4	342	11	492.6	6.19
5	231	5	313.3	16.34
6	1045	37	1245.5	20.82

**Table 4:** Performances and results of map-matching for Trajectory 1 depending on the adaptation coefficient k.

Adaptation Coeff. (k)	No. of Points Matched Correctly	No. of Points Matched	Ratio of Correct Match (RCM)	Average Positional Error (APE)
0	355	417	0.8513	0.9365
0.1	419	465	0.9011	0.9098
0.2	418	462	0.9048	0.9037
0.3	416	460	0.9043	0.9043
0.4	413	458	0.9017	0.9055
0.5	410	457	0.8972	0.9574
0.6	404	450	0.8978	0.9741
0.7	393	441	0.8912	0.9889
0.8	373	424	0.8797	0.9450
0.9	328	401	0.8180	0.9860
1	94	202	0.4653	1.3808

**Table 5:** Performance indices from map-matching by the adaptive Fréchet-distance-based method applied for Trajectories 1–6.

Trajectory	Average Matching Distance of the Ground Truth (m)	Average Fréchet Distance (m)	Performance Indices	
			RCM	APE
1	13.8124	10.9306	0.9048	0.9037
2	12.1135	8.4391	0.9125	0.6687
3	10.9858	8.8906	0.8758	0.5717
4	8.2009	6.1022	0.9645	0.4713
5	9.1461	8.7906	0.8957	0.6808
6	11.6713	9.6762	0.8602	0.6593
Average (2–6)	10.4235	8.3797	0.8910	0.6198

**Table 6:** Performance indices from map-matching by the conventional method and the basic Fréchet-distance-based method applied for Trajectories 1–6.

Trajectory	Conventional Map-Matching Method for Vehicles		Basic Fréchet-Distance-Based Method (non-Adaptive Method)	
	RCM	APE	RCM	APE
1	0.8771	0.9259	0.8855	0.9094
2	0.8909	0.6648	0.8961	0.6720
3	0.8485	0.6214	0.8457	0.5672
4	0.9429	0.6331	0.9743	0.5356
5	0.8696	0.6815	0.8820	0.6806
6	0.8425	0.7203	0.8602	0.6752
Average (2–6)	0.8682	0.6838	0.8836	0.6198

## Abbreviations

The following abbreviations are used in this manuscript:

PNS	Pedestrian Navigation Service
PND	Pedestrian Network Data
RI	Reliability Index
AR	Adaptation Ratio
RCM	Ratio of Correct Match
APE	Average Positional Error
